# Multiple odontogenic keratocysts in Ehlers–Danlos syndrome: a rare case report

**DOI:** 10.1186/s12903-021-01472-9

**Published:** 2021-03-09

**Authors:** Anna Starzyńska, Paulina Adamska, Łukasz Adamski, Aleksandra Sejda, Piotr Wychowański, Michał Studniarek, Barbara Alicja Jereczek-Fossa

**Affiliations:** 1 Department of Oral Surgery, Medical University of Gdańsk, 7 Dębinki Street, 80-211 Gdańsk, Poland; 2 Department of Pathomorphology, University of Warmia and Mazury, 18 Żołnierska Street, 10-561 Olsztyn, Poland; 3 Department of Oral Surgery, Medical University of Warsaw, 6 St. Biniecki Street, 02-097 Warsaw, Poland; 4 Department of Radiology I, Medical University of Gdańsk, 17 Smoluchowskiego Street, 80-216 Gdańsk, Poland; 5 Division of Radiotherapy, IEO European Institute of Oncology, IRCCS, 435 Ripamonti Street, 20-141 Milan, Italy; 6 Department of Oncology and Hemato-Oncology, University of Milan, 7 Festa del Perdono Street, 20-112 Milan, Italy

**Keywords:** Ehlers–Danlos syndrome, Odontogenic keratocysts, Dentigerous cyst, Paediatric dentistry, Case report

## Abstract

**Background:**

An odontogenic keratocyst is a lesion characterized by aggressive and infiltrative growth. The lesion is characterized by the existence of satellite microcysts (microtumours) and frequent recurrence (up to 30%). Ehlers–Danlos syndrome is a condition in which collagen production or its post-translational modifications are affected. Defects in connective tissues cause symptoms, which range from mild joint hypermobility to life-threatening complications.

**Case presentation:**

We present an extremely rare case of an 11-year old girl with Ehlers–Danlos syndrome and coexistence of multiple odontogenic keratocysts.

**Conclusions:**

This case shows mainly atypical or rare association between multiple odontogenic keratocysts and Ehlers–Danlos syndrome.

## Background

Odontogenic keratocyst (OKC according to WHO 2017 classification; from 2005 to 2017 classified as keratocystic odontogenic tumour, KCOT) is a benign lesion of aggressive growth, capable of infiltrating soft tissues, with frequent existence of satellite microcysts (microtumours) and common recurrences (up to 30%). It is localised mainly in the mandible (84%) with the prevalence of the angle and the ramus. Lesions localised in the maxilla (16%) present more aggressive growth compared to the mandible. Multiple odontogenic keratocysts may be a component of naevoid basal cell carcinoma syndrome (Gorlin–Goltz syndrome; GGS; ORPHA: 377). Methods of treating OKC include enucleation, curettage, marsupialization, cryotherapy, chemical cauterization and surgical resection [1–4]. In a patient with at least two OKC one should always look for other features indicating Gorlin–Goltz syndrome (Table 1). Despite diagnosis of GGS can be based on clinical criteria (two major or one major and two minor), today it should be reinforced by genetical analysis. The mutations are autosomal dominant and may be related with genes: *PTCH1* (9q22.32), *PTCH2* (1p34.1) or *SUFU* (10q24.32) [5].

**Table 1:** Diagnostic criteria of Gorlin–Goltz syndrome

	Major criteria	Minor criteria
1	Presence of more than two basal cell carcinomas (BCC) or a history of one BCC below the age of 20 years	Macrocephaly
2	OKC in the jaw (confirmed histologically)	Congenital anomalies—cleft lip-palate, coarse face, hypertelorism, frontal bossing
3	Three or more palmoplantar pits	Skeletal anomalies—Sprengel deformity, pectus deformity, syndactyly
4	Falx cerebri calcification	Radiologic anomalies—sella turcica bridging, vertebral anomalies including hemivertebra and combined vertebral corpi, flame-like lucency on hand and foot X-rays
5	Bifid or combined costae	Medulloblastoma
6	Presence of a diagnosis of Gorlin–Goltz syndrome in a first-degree relative	Ovarian fibroma

Odontogenic keratocyst is a lesion of a different nature. The development of the lesion may be mild, long-term or aggressive and rapid, infiltration of adjacent tissues and presence of satellite microcysts are also possible. The growth of OKC is usually asymptomatic, so it is often discovered incidentally during radiographic examinations performed for other reasons [6–10]. The treatment of choice should be done with complete enucleation of the lesion with possible extension of the procedure to peripheral bone curettage, decompression, marsupialisation, application of Carnoy's fluid or cryodestruction. In some cases, there are documented completed regressions of lession after marsupialisation or decompression. In histopathology examination, the lining of many decompressed cysts appeared like normal mucosa rather than odontogenic keratocyst. Decompression could have sometimes an interest in management of large lesions in children. Carnoy’s fluid is not registered in Poland, and in our case, a cystectomy with curettage was a treatment of choice. The patient with multiple OKC should have a check-up for 5 years at least once a year. The risk of relapse depends on the treatment method used: marsupialization—32.3%, enucleation—23.1%, enucleation with mechanical ostectomy—17.4%, two-staged therapy—14.6%, enucleation with cryosurgery—14.5%, enucleation with Carnoy’s solution—11.5%, bone resection—8.4% [11]. In addition, regular radiological examinations should be performed to detect possible recurrence [4].

Ehlers–Danlos syndrome (EDS) is a group of genetic disorders that mainly affect collagen production or post-translational modifications, also the intercellular matrix of connective tissue, as well as dysfunctions of glycosaminoglycans biosynthesis, the complement system and intracellular processes [12–16]. Major symptoms of most forms include skin hyperextensibility, atrophic scars and joint hypermobility. Various forms of EDS have been classified in several different ways depending on gene mutations and severity of symptoms [12–16].

Manifestations of EDS in the oral cavity are very common. However, odontogenic keratocyst is not commonly associated to EDS and especially multiples OKC were very rarely described in EDS. To our knowledge, there have been only two patients with EDS and co-existing OKC reported in the literature (Table 2) [15, 16].

**Table 2:** Review reports about EDS with odontogenic keratocyst

No	References	Sex	Age	EDS	OKC Location	Treatment	Follow-up	Outcome
1	Carret al. [14]	39	Female	Type II	Right angle and body of mandible	Enucleation	Two years	No sign of recurrence
2	Ferreira et al. [15]	15	Female	Mild type II or III	Right body of mandible	Enucleation	The patient was monitored but the exact follow-up period was not provided	No sign of recurrence

The aim of the study was to present an extremely rare case of an 11-year old girl with Ehlers–Danlos syndrome and coexistence of multiple odontogenic keratocysts.

## Case presentation

In 2013 an 11-year-old female was admitted to the Department of Maxillofacial Surgery Medical University of Gdańsk due to multiple lesions of the mandible and the maxilla detected on an orthopantomogram (OPG not available). At the age of 7 the patient was diagnosed with the classic type of Ehlers–Danlos syndrome (skin hyperextensibility, joint hypermobility [major symptoms], smooth skin, easy bruising, positive family history—father with EDS [minor symptoms]). The patient had been hospitalized twice before: in the orthopaedic ward due to Sprengel’s deformity and scoliosis and in the ophthalmological ward due to exotropia and hyperopia. No other significant information was found in the anamnesis.

**Fig. 1:**
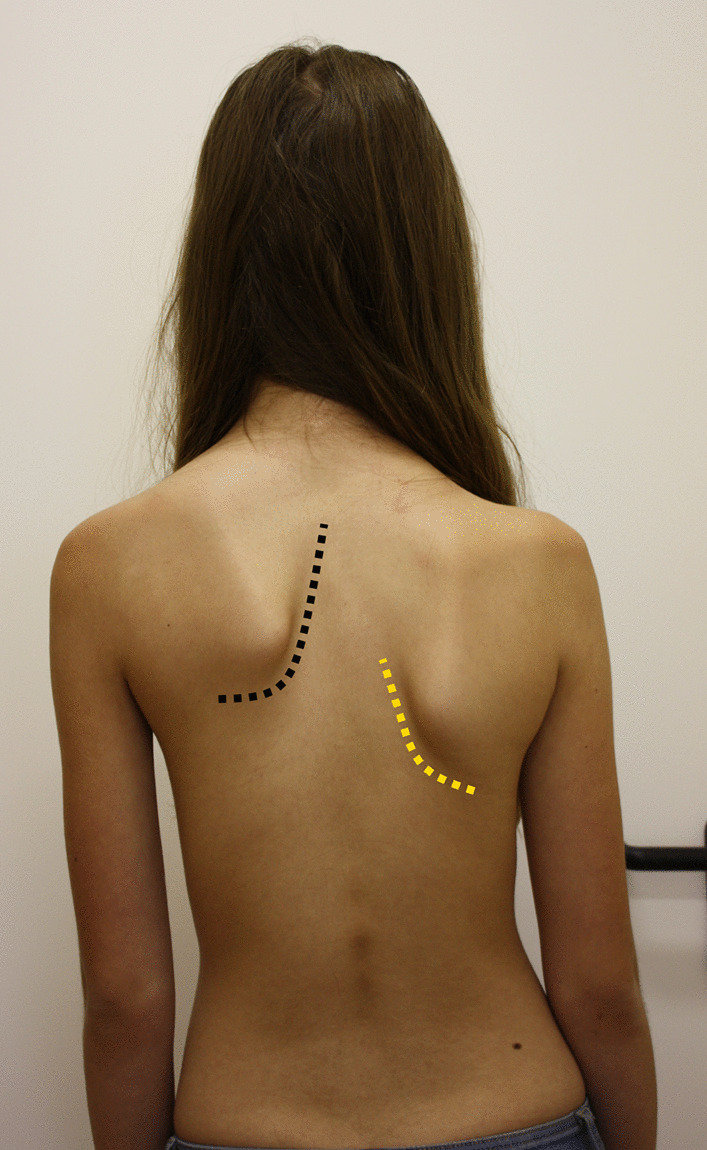
Sprengel’s deformity of left scapula (high position of scapula—black line) and correct position of the right scapula (yellow line)

The physical examination on admission revealed joint hypermobility, high scapula, thoracic spine scoliosis, skin hyperextensibility (Fig. 1), hypertelorism, exotropia and wide position of zygomatic bones. Deformation of the right ramus of the mandible and teeth displacement due to migration were observed intraorally.

Based on the OPG, multiple cysts in mandible and maxilla were suspected (radiolucent lesions circumscribed by a radiopaque halo, involving corpus, ramus and angle of mandible on both sides; radiolucent lesion around impacted right upper canine). Due to the large number of lesions, first enucleation of lesions on the right side of the mandible was planned.

The operation was performed under general anaesthesia. Intraoperatively, after muco-periosteal flap elevation, massive destruction of the buccal cortical plate and right mandibular body was observed. There was no bone support for the mesial root of the first right mandibular molar. Second right lower deciduous molar was extracted. The cysts associated with the impacted mandibular right second premolar and with the mandibular third right molar were both enucleated, the teeth involved were removed and the bone lodges were curetted. Excised tissue was submitted for histopathological examination (histopathological specimen 1, histopathological specimen 2). The wound was sutured. Post-operative healing process was uneventful.

**Fig. 2:**
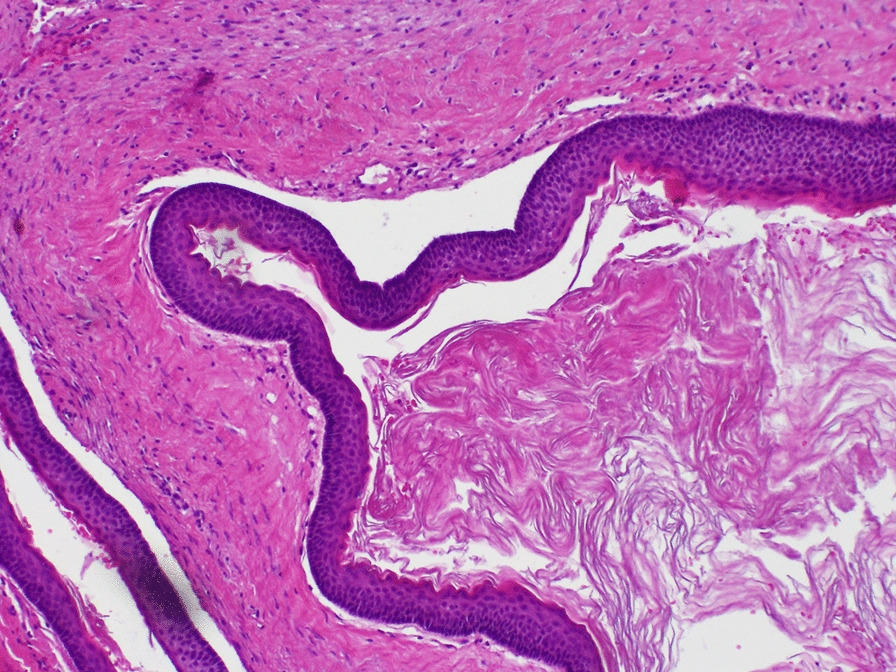
Histopathological specimen 1: corrugated keratinised stratified squamous epithelium without features of dysplasia, hyperchromatic nuclei, and palisading arrangement of basal cells (HE, magnification × 10): **a** epithelium; **b** basal cell layer

**Fig. 3:**
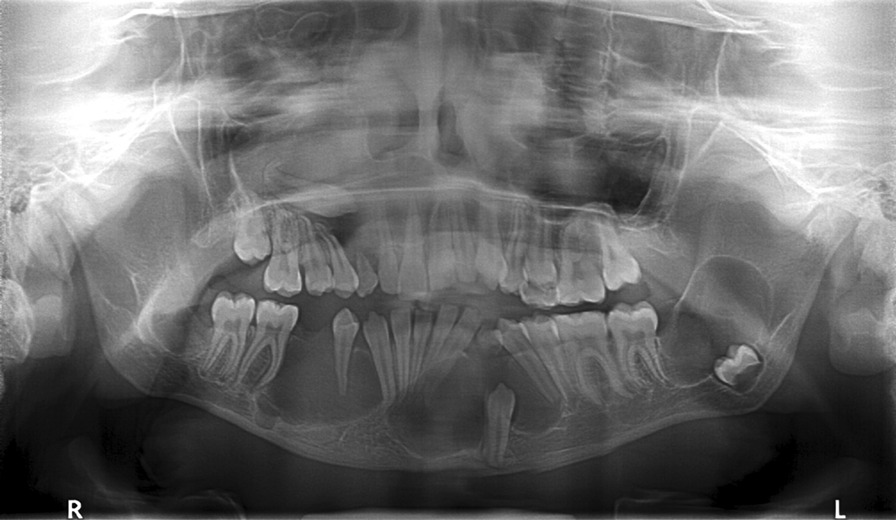
Orthopantomography—1 month after the first surgery (May 2013): radiolucent lesions circumscribed by a radiopaque halo, involving corpus, ramus and angle of mandible on both sides. Radiolucent lesion around impacted right upper canine

Histopathological examination of both tissue samples exhibited corrugated keratinised stratified squamous epithelium without features of dysplasia, hyperchromatic nuclei, and palisading arrangement of basal cells. The results confirmed the diagnosis of odontogenic keratocyst in both cases (Fig. 2). The diagnosis of Gorlin–Goltz syndrome (GGS) was established on the basis of clinical features as she had multiple odontogenic keratocysts (one main criterium), protrusion of the frontal bone, hypertelorism, scoliosis and Sprengel's deformity (four minor criterias). Diagnosis of GGS has not been extended by genetic tests. One month after the operation, normal tissue healing was observed. OPG was performed (Fig. 3). A second surgery was planned for enucleation of the others lesions.

**Fig. 4:**
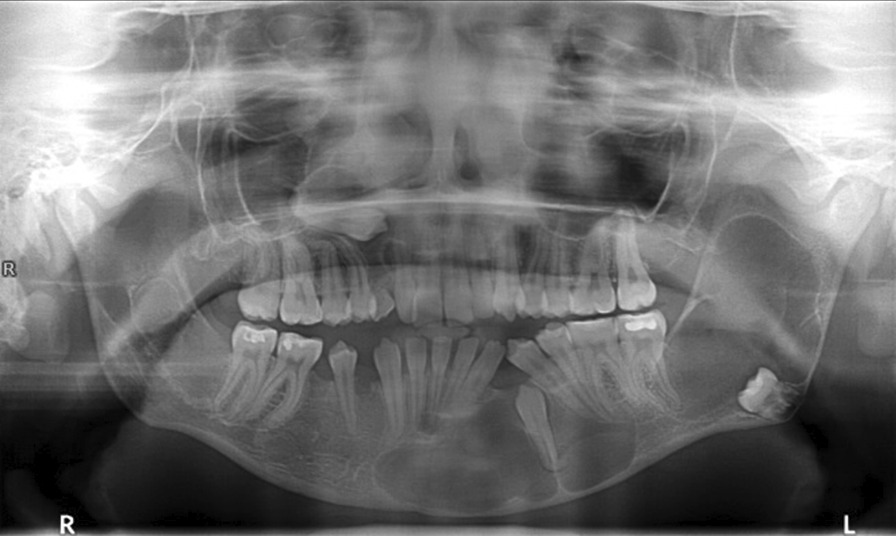
Orthopantomography—4 years after the first surgery (January 2017): enlargement of the lesions in the left mandibular body and ramus; trabeculae in the post-operative cavities

**Fig. 5:**
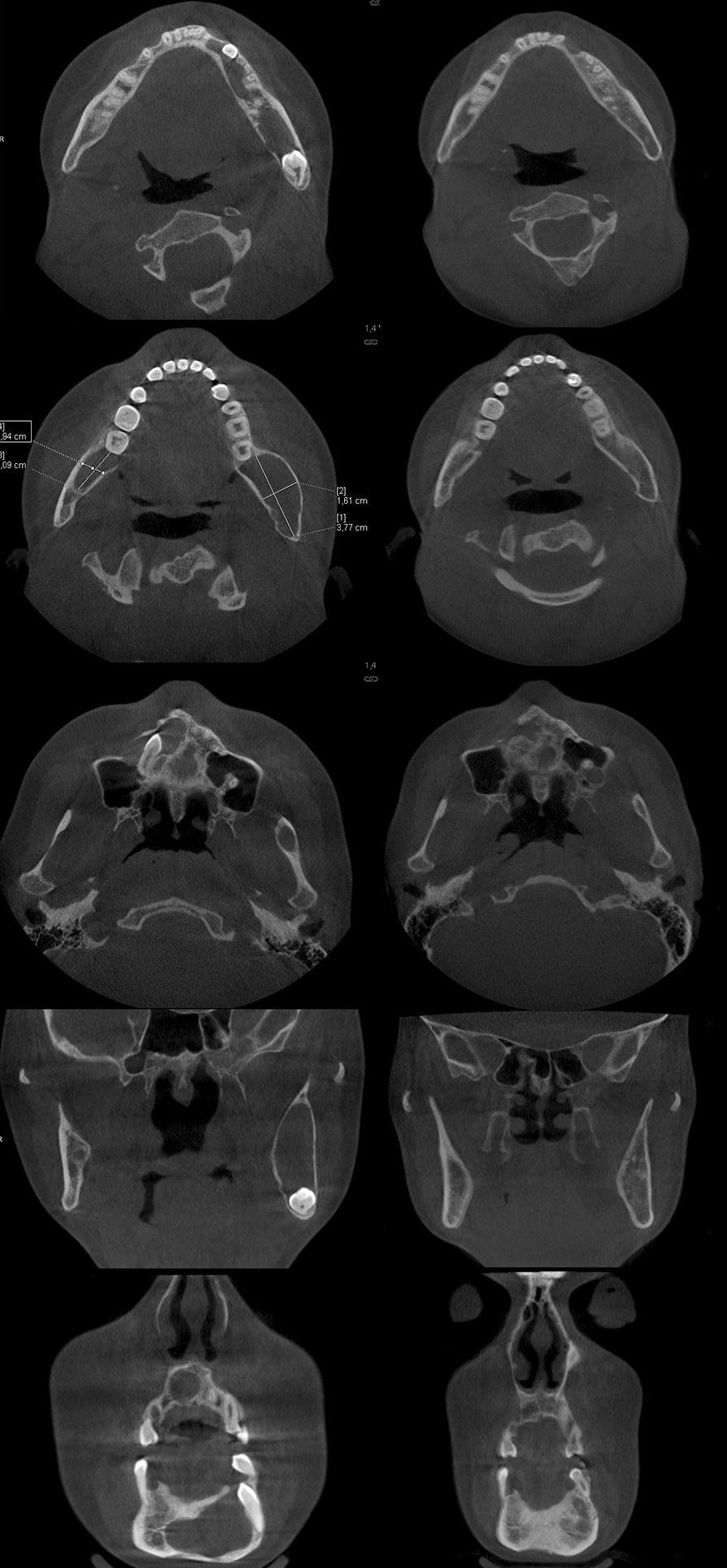
Cone beam computed tomography: **a** axial view—radiolucent lesion in left body and ramus of the mandible; **b** axial view—radiolucent lesion in right (0.94 × 2.09 cm) and left (1.61 × 3.77 cm) rami of the mandible; **c** axial view—radiolucent lesion surrounding an impacted maxillary right canine; **d** coronal view—radiolucent lesions in maxilla and mandible; **e** coronal view—radiolucent lesions in the mandible; **f** axial view—radiolucent lesion in the anterior mandible; **g** axial view—bone trabeculae in the post-operative cavity posteriorly to the mandibular right second molar; **h** axial view—radiolucent lesion after surgical removal of the impacted maxillary right canine; **i** coronal view—bone trabeculae in the post-operative cavity in the ramus; **j** coronal view—bone trabeculation in the post-operative cavity in the mandible; **a**–**e** (January 2017); **f**–**j** (October 2020)

The patient did not show up for the second operation and discontinued the treatment. Between 2013 and 2017 she had been consulted in two maxillofacial surgery clinics, but there had been no surgical treatment. The patient was readmitted in January 2017. She reported throbbing pain in the left mandibular angle and the anterior part of the mandible, as well as numbness of the chin on the right side. OPG was performed (Fig. 4) and revealed a very good bone healing in the areas of previous post-operative lodges of the right mandibular body and ramus. Cone beam computed tomography (CBCT) was performed for imaging assessment (Fig. 5a–e) and showed an enlargement of the radiolucencies located in the left mandibular body and ramus were observed. The examination did not reveal any additional information of importance.

Under general anaesthesia, the remaining mandibular cysts associated with the impacted mandibular left canine and with the mandibular left third molar were both enucleated, the teeth involved were removed and the bone lodges were curetted (histopathological specimen 3, histopathological specimen 4). Keratocystic masses were removed from the cysts. The tissue was submitted for histopathological assessment. The wound was sutured. Post-operative healing was uneventful and the patient was discharged the day after. Histopathological examination revealed odontogenic keratocysts in both samples.

**Fig. 6:**
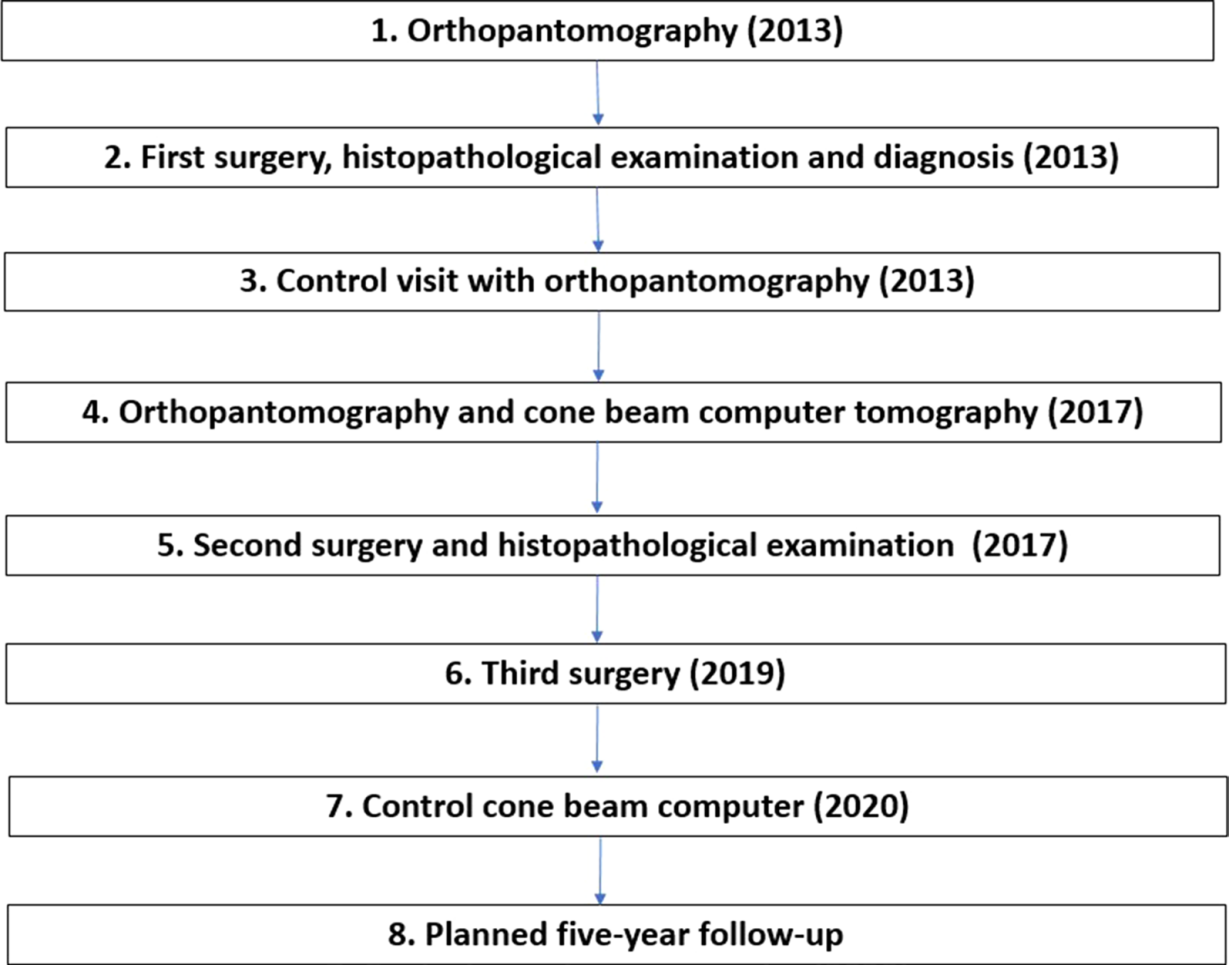
The management of the patient

The patient has been lost and showed up in October 2020 due to the presence of a purulent intraoral fistula located in the area of the lower left premolar. According to the documentation brought by the patient, she underwent another surgery in August 2019 in a different maxillofacial surgery department of the country. Enucleation of the lesion associated with the impacted maxillary right canine and lesions in the left side of the mandible were performed by others surgeons. Consecutively to this surgery, patient suffered from post-operative paraesthesia located in the nervous territory of the left inferior alveolar nerve. CBCT performed in our Hospital in October 2020 did not reveal any early recurrence (Fig. 5f–j). A follow-up for at least 5 years was planned. Figure 6 summarize the management of the patient.

## Discussion and conclusions

The Ehlers–Danlos syndrome primarily affects the synthesis of collagen, and thus the condition of the connective tissue and epithelium. The oral mucosa is friable and easily injured [17–20]. During procedures, one should pay attention to possible higher bleeding risk [17, 21–24]. Wound healing may be slower, and may progress to scarring or keloids [25–28]. *PTCH1* gene (9q22.32) for GGS and *COL5A1* gene (9q34.3) for the classic form of EDS, are both found on the same chromosome on the longer arm. Thus, there is a possible physiopathological link between GGS and EDS, from a developmental point of view with major molecular aspects [12–16]. Further studies must be undertaken to explain these exceptional associations of two rare syndromes.

Simultaneous occurrence of EDS and odontogenic keratocyst is an extremely rare finding, being reported in the literature only twice. First was described by Carr et al. in 1988 and it was a case of a 39-year old woman with type II EDS and OKC located in the right angle and the body of mandible. The only other description was the one of a 15-year old girl with type II or III EDS and OKC, described by Ferreira et al. in 2008 [15, 16]. Interestingly, both described patients were female and the lesions were located in the mandible and in both cases enucleation was the treatment of choice. In our patient, OKC was found in the maxilla as well. Unlike the patients from the mentioned reports, our patient presented with the recurrence of OKC after the surgical treatment. Since our patient was treated in more than one medical centre, assessment of the treatment is rendered difficult. Treatment of an adolescent patient is also challenging due to the fact that the development is not yet complete. In case of multiple lesions, which is a feature of Gorlin–Goltz syndrome, one-stage procedure is not always possible. Moreover, if many teeth are associated with the lesions, the decision about their removal should be considered carefully. Minimising the risk of recurrence is of utmost importance, yet one should also bear in mind the psychological impact of the loss of dentition on a young patient. Long treatment, involving multiple visits requires a disciplined patient. Adolescents and their parents should be made aware of the importance of the condition and the treatment, psychological care should be provided. Especially in patients suffering from multiple conditions.

The patient with multiple OKC should have a check-up for 5 years at least once a year. In addition, regular radiological examinations should be performed to detect possible recurrence. In the meantime, interdisciplinary approach is required for diagnosis and to find the best therapeutic strategy in these complex genetic diseases.

## Data Availability

All data and material supporting our findings are contained within the manuscript.

## Abbreviations

CBCT: Cone beam computed tomography
KCOT: Keratocystic odontogenic tumour
OKC: Odontogenic keratocyst
